# Performance Analysis of an Aperture-Coupled THz Antenna for Diagnosing Breast Cancer

**DOI:** 10.3390/mi14071281

**Published:** 2023-06-22

**Authors:** Anupma Gupta, Vipan Kumar, Dinesh Garg, Mohammed H. Alsharif, Abu Jahid

**Affiliations:** 1 Department of Interdisciplinary Courses in Engineering, Chitkara University Institute of Engineering and Technology, Chitkara University, Rajpura 140401, India; anupmagupta31@gmail.com; 2 Department of Electronics and Communication Engineering, Sri Sai College of Engineering and Technology, Pathankot 145001, India; er.vipingupta14@gmail.com; 3 Department of Computer Science Engineering, Sri Sai College of Engineering and Technology, Pathankot 145001, India; 4 Department of Electrical Engineering, College of Electronics and Information Engineering, Sejong University, 209 Neungdong-ro, Gwangjin-gu, Seoul 05006, Republic of Korea; 5 School of Electrical Engineering and Computer Science, University of Ottawa, 25 Templeton St., Ottawa, ON K1N 6N5, Canada

**Keywords:** aperture-coupled, breast cancer, THz imaging technology, polyamide

## Abstract

The most important technique for exposing early-stage breast cancer is terahertz imaging. It aids in lowering the number of breast cancer-related fatalities and enhancing the quality of life. An essential component of developing the THz imaging system for high-quality photos is choosing the right sensor. In this article, a wideband antenna for microwave imaging of breast tissue with an operating frequency of 30 GHz (107 GHz to 137 GHz) is constructed and analyzed. An aperture-coupled antenna with an optimized ground aperture is proposed and analyzed, which made it possible to obtain better and consistent impedance matching in the wideband spectrum. The variation of backscattered signal energy in body tissue is assessed with healthy breast tissue and in the presence of malignant cells. A significant difference in energy scattering is observed for both situations. The suggested antenna’s linear and stable time domain characteristics make it an appropriate component for THz imaging technology.

## 1. Introduction

### 1.1. Background and Motivation

Terahertz imaging is growing rapidly as a noninvasive technology for the detection of unhealthy and cancerous cells in breast tissue. THz imaging is a non-ionizing and low-power technology, making it biologically hazardous and free for in vitro and in vivo operations. One THz frequency requires an energy level of about 4.14 meV, whereas the minimum X-ray energy level is 0.12 Kev. Therefore, THz imaging is safe and reduces the ionization effects as compared to conventional methodologies. This non-ionizing behavior is an important and critical feature that advances THz systems in healthcare applications [1]. High-frequency THz electromagnetic radiation has a fundamental time period of about 1-ps, which makes the biological investigation system faster and limits the thermal effects on healthy tissue. Due to shorter wavelengths, THz frequencies have a superior imaging resolution and have enhanced transmission properties in thick and fatty tissues. The electromagnetic spectrum for the terahertz frequency range is generally expected from 0.1 THz (100 GHz) up to 10 THz [2].

In THz imaging systems, variations in water contents of healthy and unhealthy cells are analyzed to diagnose the existence of specific cancer cells and to measure the depth, location, and severity of damaged tissues. Unhealthy tumors and cancer cells have high water levels, and THz photons are highly sensitive to water absorption. It helps in the characterization of THz radiation in biological and medical imaging [3]. Pulsed THz radiation can sense very small cancer cells and has the potential to differentiate between healthy and cancer cells. THz imaging is useful for quick imaging during surgery, with clear differentiation between peritumoral and cancerous cells, and provides accurate boundary margins (thickness between cancer and normal cells) to the surgeon to aid in the removal of unhealthy cells [4]. The THz radiation source is the most significant part of the THz biomedical imaging system [5,6].

### 1.2. Related Works

Antennas with small planar geometry, high directivity, broad bandwidth, and on-chip fabrication capabilities have the potential to be a promising component of the imaging system as a THz radiation source. Photoconductive antennas (PCA) are the most commonly used antennas for biomedical imaging in the THz range. A high-resistance thin film of semiconductor materials such as gallium arsenide (GaAs) or zinc oxide (Zno) with two metallic contacts is used to design the PCA [7,8]. Microstrip patch antennas with transparent radiators (consisting of indium tin oxide) and low-resistance materials (such as graphene) are also gaining research interest as a practical alternative to match the coming requirements of the medical healthcare industry [9,10]. Metamaterial technology is incorporated with THZ patch antennas to enhance the radiation characteristics such as efficiency, directivity, and gain [11,12,13,14].

Arraying of planar and patch antennas is used to improve the gain [15,16]. However, high resolution is the most important characteristic of imaging applications, which can be gained through a broad bandwidth. In order to achieve a wider frequency spectrum, various types of antenna structures are proposed for a wider frequency spectrum for THz medical imaging, for example, the Yagi-Uda antenna is designed [17] with a 27% bandwidth. Fractal geometry [18], a multiband spectrum [19], aperture coupling [20], MIMO with reconfigurable structures [21], and PBG and SIW technology-based graphene antenna [22] are the different planar antenna technologies for a wider bandwidth. However, the best existing THz antenna structures possess lower bandwidth and poor far-field characteristics. They show hitches to be practically implemented in medical imaging. For the easiness of understanding and readability, a brief of some THz antenna structures is depicted in Table 1. Apart from this free space antenna, studies have shown that layered structures, graphene-based technology, and metamaterial technology have excellent effects on radiating characteristics [23,24,25]. In this work, a layered structure is used for breast cancer detection application in the THz region.

### 1.3. Contributions

The present study introduces and investigates an aperture-coupled THz antenna with a polyamide substrate for the purpose of breast cancer detection in medical imaging. Drawing from the relevant literature, THz imaging is based on the principle that changing the electrical properties of bodily tissue leads to alterations in the backscattered parameters. Cancerous tissue, possessing a higher dielectric constant than normal breast tissue due to its high water content, is the focus of an investigation, with the deviation of the S11 signal being analyzed. The antenna developed in this study contributes to the field in the following ways:

(1) It features a planar and straightforward structure that can be easily traced onto non-planar breast tissue.
(2) Its wider impedance bandwidth allows for radiation penetration into tissue at different depths.
(3) It offers higher gain.
(4) It displays good sensitivity to unhealthy tissue.

### 1.4. Paper Organization

The paper is divided into the following sections: the introduction and literature review are found in Section 1. The proposed aperture-coupled antenna’s design technique is presented in Section 2. The proposed experiment setup, together with its outcomes and analysis, are presented in Section 3.

## 2. Antenna Topology and Performance Analysis on Free Space

**Figure 1:**
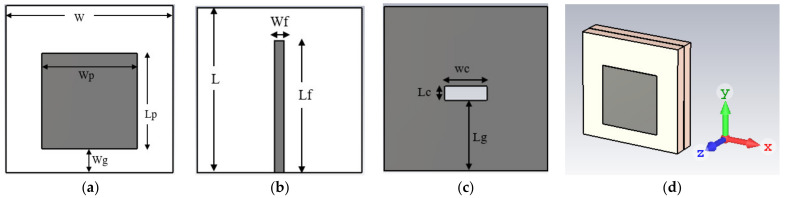
Topology of the different parts of the proposed aperture-coupled structure: (**a**) uppersubstrate printed with a radiator, (**b**) bottom substrate with a feedline, (**c**) ground plane with the aperture below the bottom substrate, and (**d**) perspective view.

**Figure 2:**
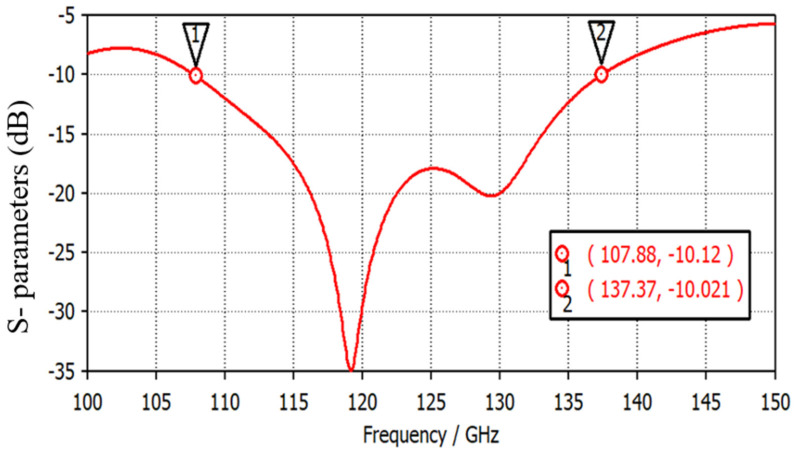
The |S11| parameter of the proposed antenna.

**Figure 3:**
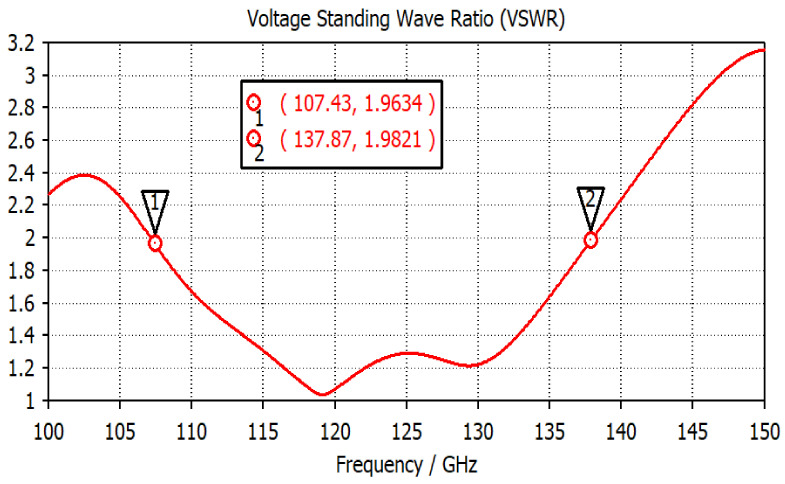
The VSWR of the proposed antenna.

An aperture-coupled THz antenna is operated over a wide-frequency spectrum of 30 GHz (from 0.107 THz to 0.137 THz). In the aperture-coupled structure, a metallic ground plane is sandwiched between the two dielectric substrates. The radiating element is designed on the upper side of the first substrate layer, and the microstrip feed line (to excite the radiator) is designed on the lower surface of the second substrate layer. To generate the coupling effect between microstrip line and radiating element, a rectangular slot (working as the aperture) is engraved in the ground. The antenna is designed using a low-permittivity dielectric substrate polyamide of permittivity (ε_r_) of 3.5. The configuration of the designed THz antenna is represented in Figure 1. The topological parameter values of the proposed design are given in Table 2. The surface area of the antenna is 3.5 mm × 3.5 mm. The power is coupled from the microstrip line to radiating patch through the rectangular aperture on the ground layer. The position and width of the aperture are optimized to achieve the maximum impedance matching and −10 dB bandwidth. The |S11| parameter for the proposed antenna with a rectangular aperture is represented in Figure 2. The antenna shows a −10 dB impedance bandwidth from 0.107 THz to 0.137 THz. A wide bandwidth of 30 GHz is suitable to achieve good resolution for breast cancer imaging. The VSWR plot in Figure 3 represents the impedance matching of the antenna over the operating frequency band. A VSWR value less than two is desired for the impedance matching.

**Figure 4:**
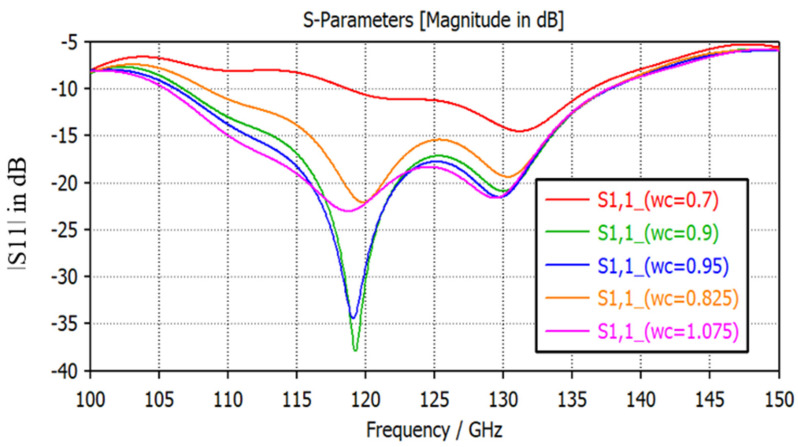
The |S11| parameter for the varying slot widths (wc).

The size and position of the aperture are the most important design parameters of aperture-coupled structures. Coupled power varies the resonance current and resistivity of the patch and stimulates the multiple resonance mode, which helps to attain the broader bandwidth. The position of the aperture is centered below the patch, as it provides the dominant magnetic polarization and maximum coupling effect. Hence, it is required to adjust the size of the aperture. The size of the coupling aperture should be smaller than the radiator to agree with the maximum impedance matching, lower back scattered power, and enhanced efficiency [21]. As the area of the aperture slot is increased, the coupling effect is enhanced, but it leads to an increase of the antenna impedance. In turn, it causes the deterioration of impedance matching [22]. Thus, to gain the optimum impedance matching and broader bandwidth, the width (‘wc’) of the aperture slot is varied and analyzed through a parametric sweep. The plot for varying the width reflection coefficient is shown in Figure 4. The slot width is varied from 0.7 mm to 1.0 mm. Optimum coupling is achieved at the width ‘wc’ of 0.9 mm. The bandwidth for the lower cut-off frequency increases with the increasing slot width but reduces the impedance matching.

**Figure 5:**
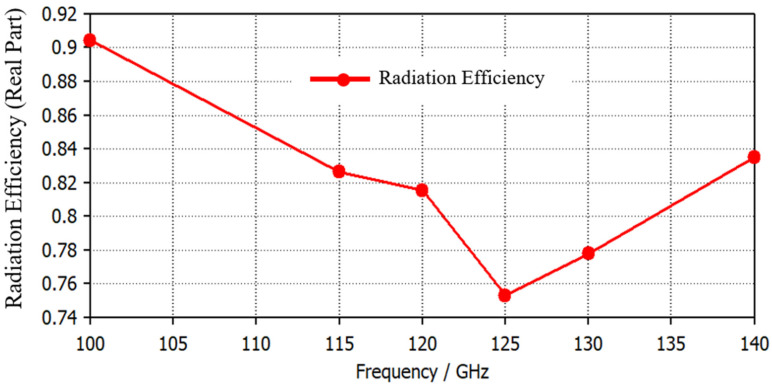
The radiation efficiency of the antenna.

**Figure 6:**
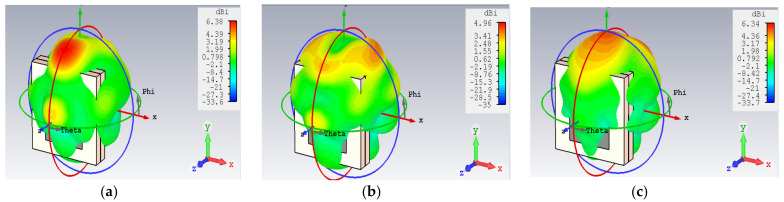
The gain of the antenna (**a**) at 115 GHz (**b**), 125 GHz (**c**), and 135 GHz.

The radiation efficiency of the antenna over the entire frequency spectrum is plotted in Figure 5. The antenna shows more than a 75% radiation efficiency. A 3D power pattern is represented in Figure 6. It can be observed that gains of 6.38 dBi at 115 GHz, 4.96 dBi at 125 GHz, and 6.34 dBi at 135 GHz are obtained. The maximum power is oriented towards the front lobes, representing the low power loss in the back and side lobes. The wide bandwidth and efficient radiation characteristics of the proposed antenna make it an appropriate candidate for THz imaging.

## 3. Performance Analysis of Antenna for Breast Cancer Detection

### 3.1. Design of Breast Phantom and Simulation Setup for Imaging of Breast Cancer

**Figure 7:**
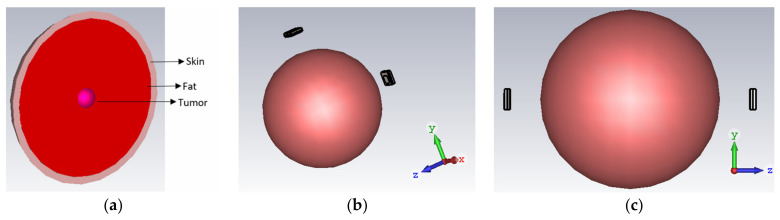
(**a**) Breast tissue with a tumor. (**b**) Simulation setup for cancer detection: a side-by-side view and (**c**) a face-to-face view.

The definitive objective of the proposed structure is to extricate the unhealthy tissues inside a breast by detecting the dissimilarity in the |S11| signal (backscattered signal) of the antenna between healthy and unhealthy tissues. In order to accomplish this, an equivalent breast tissue phantom in a spherical shape with a 20 mm radius (average value) is considered. It consists of a 2 mm skin layer (ε_r_ = 38 and conductivity = 1.49 S/m), 16 mm of a fat layer, a tumor radius of 2 mm, ε_r_ = 67, and conductivity of 47 S/m [26,27]. The designed phantom model, along with the simulation setup, are shown in Figure 7a. To accumulate the antenna backscattered signal, two setups are considered. Figure 7b,c depict two antenna structures arranged in a face-to-face position with a 1 mm separation between each antenna and the tissue.

### 3.2. Backscattered Signal Analysis for Tumor Detection

**Figure 8:**
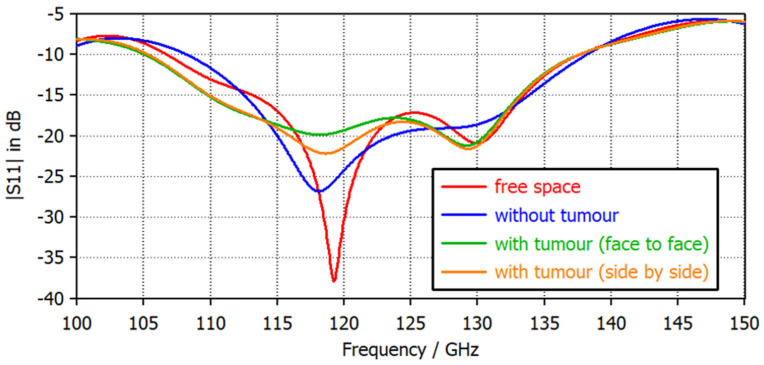
Backscattered signal of the proposed antenna.

According to the literature, unhealthy tissue has a higher water content and higher relative permittivity as compared to healthy tissue. Thus, at the interface of two diverse mediums, electromagnetic radiation will undergo multiple reflection and scattering effects. Figure 8 represents the backscattered signal of the antenna for the free space, without tumor, and with tumor (side-by-side and face-to-face) configurations. In the free space, the antenna has enclosed the frequencies from 0.107 THz to 0.137 THz; that is significant for the dispersion of radiated power at different depths. As the antenna is positioned corresponding to the tissue (healthy breast), a large difference in the |S11| parameters (backscattered signals) can be noticed over the whole frequency spectrum. Similarly, when placing the antenna on the cancerous breast tissue, a dissimilarity in the scattering parameters can be detected. For both the evaluation setups, it can be observed that the maximum deviation of the scattering signal is obtained at 118 GHz. For the face-to-face configuration, there is a deviation of 7 dB, and for the side-by-side configuration, a deviation of 5 dB is found. The entire operational band’s |S11| displays substantial change, which qualifies the antenna to detect the presence of tumor cells. Further, a summary of the variations that happen in the value of the reflection coefficient at different frequencies of the transmitted THz pulse is shown in Table 3.

**Figure 9:**
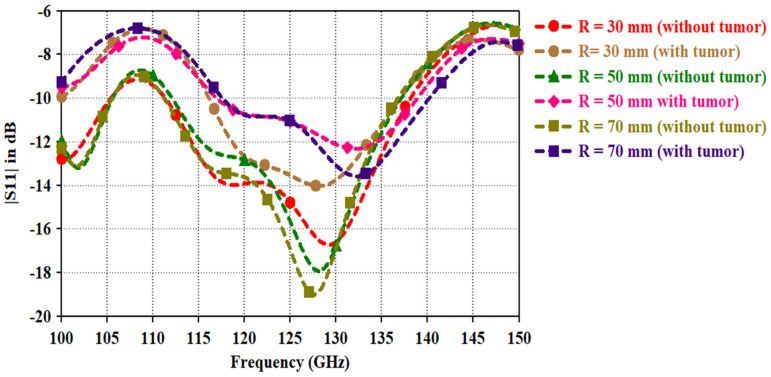
Backscattered signal for varying phantom sizes with the radius (R).

As studied in the literature [28,29,30], breast tissue includes different dielectric layers with varying thickness. Variations in the dielectric materials have a significant impact on the antenna performance and sensitivity to tumors. Thus, an analysis of the backscattered signal for varying breast sizes of the radius R with a tumor and without a tumor is performed. Figure 7a depicts a phantom model with a fat layer. In the case of a 70 mm spherical phantom, the skin layer has a thickness of 3 mm, the fat layer has a thickness of 62 mm, and a 5 mm tumor is present. For the 50 mm phantom, the skin layer thickness is 2.8 mm, the fat layer radius is 42.2 mm, and the tumor measures 5 mm. In the case of a 30 mm phantom, the skin thickness is 2.5 mm, the tumor radius is 4 mm, and the fat layer has a radius of 23.5 mm. The reflection coefficient for varying the phantom sizes is shown in Figure 9. It is evident that, as the phantom size increases, the backscattered signal shifts upwards to 125 GHz and downwards towards higher frequencies. With a tissue size of 70 mm, the antenna covers the entire bandwidth spectrum from 112 GHz to 135 GHz, exhibiting significant deviations in the reflection coefficient in the presence of unhealthy tumor cells within the breast. For a radius of 70 mm, a maximum deviation of 7 dB is observed at 127 GHz, while, for a radius of 30 mm, a maximum deviation of 4 dB occurs at 117 GHz. These findings underscore the impact of breast size and tumor presence on the backscattered signal. The results demonstrate the ability of the antenna to detect and differentiate tumor-related deviations in the reflection coefficient within specific frequency ranges. Such insights contribute to the understanding and development of efficient antenna systems for terahertz imaging in breast cancer detection.

**Figure 10:**
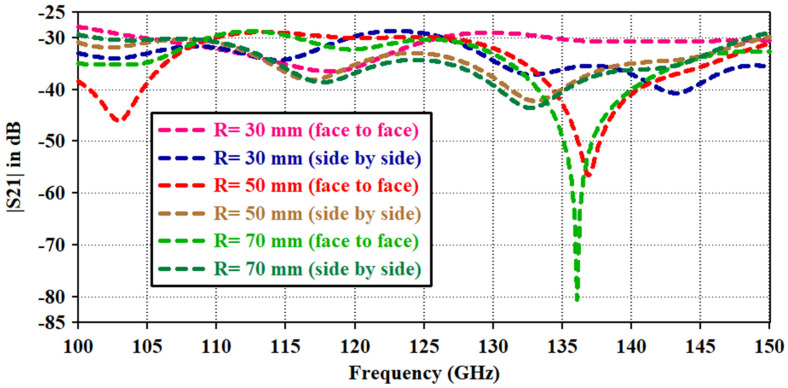
Transmission coefficient (|S21|) of the antenna.

**Figure 11:**
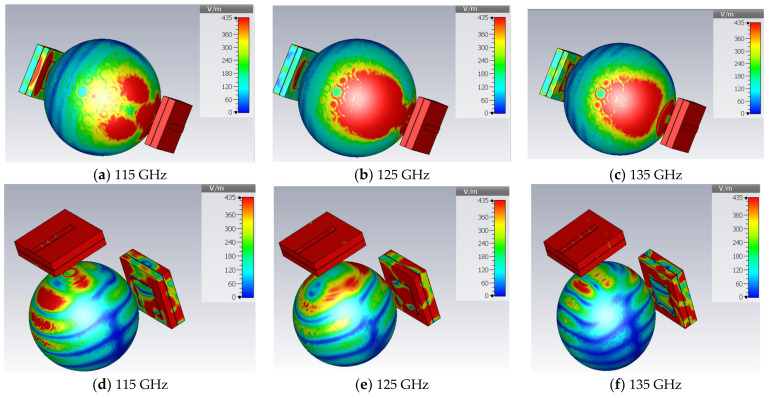
Electric field distribution for a face-to-face (**a**–**c**) and side-by-side (**d**–**f**) configuration.

Figure 10 shows the transmission coefficient |S21| of the antenna for the two matching antennas in a side-by-side and face-to-face configuration for different phantom sides. |S21| is examined to take the far-field effect of the two antennas on each other over the whole bandwidth. It should be low, as it indicates a direct power transfer from one antenna to another. The |S21| value is below −30 dB for all the phantom sizes in both the side-by-side and face-to-face configurations. Except at 137 GHz in the face-by-face configuration, a stable |S21| is accomplished in both operational scenarios. The transmission coefficient exhibits a similar type of volatility as shown in [21]. The electric field distribution is measured and depicted in Figure 11 to support the fluctuation of the power distribution in heterogeneous body tissues. It is obvious that the power absorbed by the tissue layers is not uniform, and some of the absorbed power is taken up by the tumor, which causes deflections in the radiation patterns coming from various ray directions.

**Figure 12:**
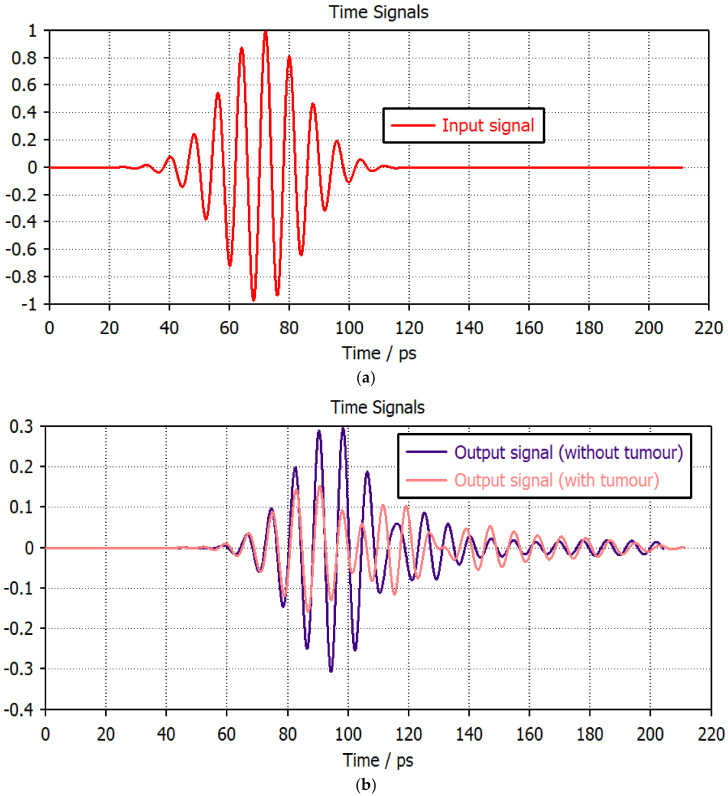
(**a**) Transmitted and (**b**) received pulses for the face-to-face setup.

**Figure 13:**
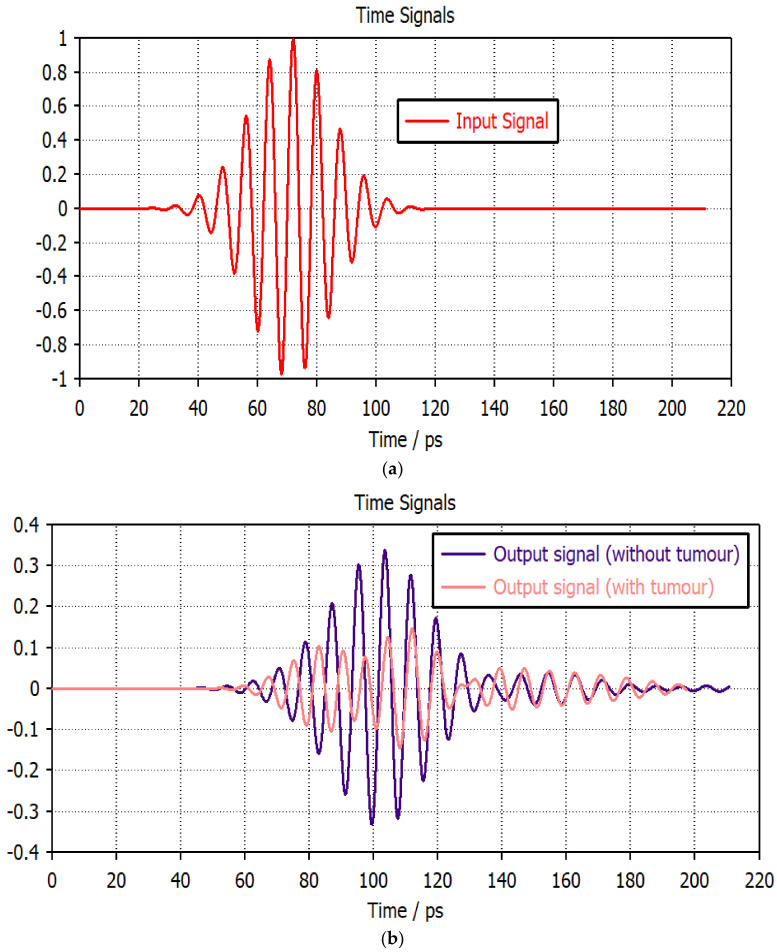
(**a**) Transmitted and (**b**) received pulses for the side-by-side setup.

Although the antenna’s frequency domain performance is consistent, imaging requires research into the time domain performance. Figure 12 and Figure 13 display the input signal and the received signal for the two evaluation setups with and without a tumor. For all configurations, the received waveform is aligned with the transmitted signal with minimal variations. As a result, the convenience of an antenna for THz imaging is represented by both a time and frequency domain analysis. In Table 4, a comparison of the proposed THz antenna for imaging applications with the existing structures is listed.

The provided Table 4 presents a comparison of various studies on terahertz imaging for cancer evaluation, highlighting the frequency, size, bandwidth, gain, evaluation parameters, and deviation values. It offers insights into different approaches and their performances in detecting and evaluating cancerous conditions. Reference [9] reported a frequency of 0.312 THz, but no bandwidth was specified. The evaluation parameter and deviation value were not provided, making it challenging to assess the effectiveness of the approach. Similarly, References [11,12] provided specific sizes and gains, but the evaluation parameters and deviation values were not mentioned. This lack of information limits our understanding of their effectiveness in cancer evaluation. Reference [13] focused on a frequency of 0.198 THz, with a size of 600 × 600 µm^2^. The evaluation parameters included the reflection coefficient and E-field, and a deviation value of 3 dB was reported for a 200 µm tumor. Reference [16] provided a frequency of 0.132 THz and a size of 500 × 960 µm^2^. While the gain was given as 5.6 dB, the evaluation parameters and deviation values were not analyzed or reported. Thus, it is challenging to determine the performance of this approach in cancer evaluation. Reference [20] explored a frequency of 0.7 THz and a size of 300 × 300 µm^2^. The evaluation parameters included the reflection coefficient and Q-factor, with a reported deviation value of 7 dB. Reference [22] examined a higher frequency of 4.6 THz with an unspecified size. The resonance frequency and SAR (specific absorption rate) were evaluated, reporting a deviation of 0.0395 THz. This demonstrated the capability of the method in detecting frequency variations associated with cancerous tissues. This work focused on a frequency of 0.120 THz and a size of 3.5 mm × 3.5 mm. The evaluation parameters included the reflection coefficient and E-field, with a reported deviation value of 7 dB for a 2 mm radius tumor. This indicated the sensitivity of the proposed approach in detecting tumors within the specified size range. This study and Reference [13] considered the reflection coefficient and E-field. However, Reference [13] additionally analyzed the backscattered signal’s deviation value for a specific tumor size of 200 µm, reporting a 3 dB deviation. In comparison, the current study presents a deviation value of 7 dB for a 2 mm radius tumor. In terms of bandwidth and gain, Reference [13] specified a bandwidth of 15 GHz and a gain of 3.4 dBi. In contrast, the current study reports a bandwidth of 32 GHz and a gain of 6.38 dBi. These parameters affect the system’s ability to capture a wide range of frequencies and enhance the received signal.

Ultimately, with the exception of References [11,12], aperture-coupled technology exhibits a higher gain compared to simple planar geometry. However, a sensitivity analysis for unhealthy tissues was not performed in these references. Additionally, the designed structures had wider bandwidths, except for References [16,22]. Nevertheless, the proposed structure demonstrates a significantly higher sensitivity in diagnosing unhealthy cells. Furthermore, the designed structure is simple, cost-effective, and offers better gain, along with excellent sensitivity, when detecting small-sized tumors.

## 4. Conclusions

The configuration employed for the aperture-coupled patch antenna follows a standard design, which is specifically chosen to achieve two main objectives: a broad −10 dB impedance bandwidth and consistent operating characteristics across a wide range of frequencies known as ultra-wideband (UWB) frequencies. This is accomplished by utilizing a rectangular patch as the main element of the antenna, along with an optimized coupling slot that facilitates the desired performance. To assess the antenna’s effectiveness in the terahertz (THz) range, an equivalent tissue phantom is used as the testing medium. The tissue phantom serves as a representative model for the evaluation of the antenna’s performance. When the antenna is positioned on heterogeneous tissue, it exhibits a notable ability to differentiate the backscattered signal. This means that the antenna is capable of distinguishing between different signals reflected by various types of tissues. It is worth noting that the electrical characteristics of the tissue tend to change with the variations in the magnitude of the backscattered signal. This property can be effectively utilized to obtain imaging information. By analyzing the variations in the |S11| parameter, which quantifies the reflection coefficient of the antenna, significant differences can be observed. Specifically, in a face-to-face scenario at 125 GHz, there is a maximum deviation of 5 dB, while, in a side-by-side scenario at the same frequency, the maximum deviation is 7 dB. These deviations indicate the potential for acquiring valuable information through the backscattered signal. Moreover, the distribution of the electric field generated by the antenna is assessed to evaluate the presence of a nonuniform power distribution. This analysis helps to determine if there are any areas where the power concentration is significantly imbalanced, which could affect the antenna’s overall performance. Additionally, the linearity of the timing signals is evaluated using the received and transmitted pulses. By examining the timing characteristics of these pulses, it is possible to determine whether the antenna operates linearly and accurately in terms of timing. This assessment is crucial to ensure reliable and precise signal transmission and reception. In summary, the configuration of the aperture-coupled patch antenna is carefully designed to achieve a broad impedance bandwidth and stable performance at UWB frequencies. By utilizing an equivalent tissue phantom, the antenna’s performance in the THz range was evaluated, particularly in terms of its ability to differentiate backscattered signals. The variations in the electrical characteristics of the tissue, caused by changes in the backscattered signal magnitude, can be exploited for imaging purposes. The analysis of the |S11| parameter reveals significant deviations, allowing for valuable information extraction. The distribution of the electric field and the linearity of timing signals were also examined to assess the power distribution and signal timing accuracy, respectively.

## Figures and Tables

**Table 1:** Summary of the design methodology of investigated antennas for THz imaging.

Refs.	Type	Technique	Performance Enhancement Factor	Limitation
[7]	*Photo Conductive Antenna* *(PCA)*	Dipole with GaAs substrate	Wide spectrum (120 GHZ) and higher directivity	Not analyzed for application
[8]		Bow-Tie structure with plasmonic gratings by etching widows in substrate	Reduce leakage current	Not analyzed for application
[9]	*Microstrip Patch* *antenna*	array with inset feed	improved gain and beam width	performance not evaluated for time domain response
[10]		Graphene and reconfigurable patch	characterized for on-chip application	application based performance not evaluated
[16]		Array of slotted patch radiator	higher gain	Not evaluated for practical application and omnidirectional radiation pattern
[11]	*Metamaterial*	Metamaterial with defected ground	miniaturization	analyzed for breast cancer detection, only for front-to-front configuration
[12]		CSIR loaded structure	Miniaturization and increasing the resonance frequency	time domain analysis not performed and narrow bandwidth
[13]		Slotted patch with PBG	Improved reflection properties	Sensitivity very low for cancer cell
[17]	*Yagi-Uda*	PBG gratings	Size reduction and bandwidth enhancement	application based performance not evaluated
[20]	*Circular radiator*	4-graphene arc loaded	Reducing Q-factor to enhance bandwidth	Low gain
[22]	*SIW*	Graphene and PBG	Wider bandwidth	Sensitivity very low for cancer cell

**Table 2:** Geometrical values of the designed antenna.

Parameter Name	Size (mm)	Parameter Name	Size (mm)
W	3.5	L	3.5
Wp	2	Lp	2
Wg	0.5	Lg	1.5
Wc	0.9	Lc	0.3
Wf	0.2	Lf	2.8

**Table 3:** Comparison of |S11| variations in the absence and presence of tumor w.r.t. (Figure 8).

Frequency at which |S11| is Analyzed (GHz)	|S11| on Breast Tissue without Tumor (dB)	|S11| on Breast with Tumor (dB) in Face-to-Face Configurations	|S11| on Breast with Tumor (dB) in Side-by-Side Configurations	Difference in Magnitude of |S11| with and without Tumor (Face-to-Face)	Difference in Magnitude of |S11| with and without Tumor (Side-by-Side)
119	−26.5	−20	−22	6.5 dB	4.5 dB
130	−18	−21.5	−21.5	3.5 dB	3.5 dB

**Table 4:** Comparison of the proposed THz imaging antenna with existing structures.

Refs.	Frequency (THz)	Size	Bandwidth	Gain	Cancer Evaluation Parameter	Deviation Value
[9]	0.312	1105 × 500 × 100 (µm^3^)	-	6.04 dB	Not performed	-
[11]	1.00	800 nm × 800 nm	30 GHz	20 dBi	E-field	-
[12]	1.5	480 nm × 480 nm	0.3 THz	24 dBi	E-field	-
[13]	0.198	600 × 600 (µm^2^)	15 GHz	3.4 dBi	Reflection coefficient and E-field	3 dB for 200 µm tumor
[16]	0.132	500 × 960 (µm^2^)	58 GHz	5.6 dB	Not analyzed	-
[20]	0.7	300 × 300 (µm^2^)	34 GHz	2.07 dB	Reflection coefficient and Q-factor	7 dB
[22]	4.6	-	1.5 THz	5 dB	Resonance Frequency and SAR	0.0395 THz frequency deviation
This work	0.120	3.5 mm × 3.5 mm	32 GHz	6.38 dBi	Reflection coefficient and E-field	7 dB for 2 mm radius tumor

## Data Availability

Not applicable.
